# Spectrum-Effect Relationship Between Antioxidant and Anti-inflammatory Effects of Banxia Baizhu Tianma Decoction: An Identification Method of Active Substances With Endothelial Cell Protective Effect

**DOI:** 10.3389/fphar.2022.823341

**Published:** 2022-01-24

**Authors:** Nan Xu, Mingchen Li, Ping Wang, Shuling Wang, Haiyan Shi

**Affiliations:** ^1^ Laboratory of Chinese Medicine Preparation, Shandong Research Academy of Traditional Chinese Medicine, Jinan, China; ^2^ Department of Clinical Pharmacy, The First Affiliated Hospital of Shandong First Medical University & Shandong Provincial Qianfoshan Hospital, Shandong Engineering and Technology Research Center for Pediatric Drug Development, Shandong Medicine and Health Key Laboratory of Clinical Pharmacy, Jinan, China; ^3^ School of Pharmacy, Hangzhou Normal University, Hangzhou, China

**Keywords:** banxia baizhu tianma decoction, spectrum-effect relationship, antioxidant—phytochemical studies, anti-inflammatory, active substances from natural resource

## Abstract

Banxia Baizhu Tianma decoction (BBTD), a six-herb Chinese medicine formula first described approximately 1732 AD, is commonly prescribed for Hypertension with Phlegm-dampness Stagnation (HPDS) as an adjuvant therapy in China. Obesity is an important risk factor for the increasing prevalence of hypertension year by year in China. In Traditional Chinese medicine, obesity is often differentiated as the syndrome of excessive phlegm-dampness.Vascular endothelial cell injury plays an important role in the development and occurrence of HPDS. In this study, the protective effects of 18 batches of BBTD samples from different origins on HUVEC cells were evaluated, including antioxidant and anti-inflammatory activities. Ultrahigh performance liquid chromatography (UPLC) was used to establish fingerprints, and combined with pharmacodynamic indexes, the protective components of BBTD on endothelial cells were analyzed. Antioxidant and anti-inflammatory activities were evaluated by ROS and Hs-CRP models, respectively. Hierarchical cluster analysis (HCA) and Bivariate correlation analysis (BCA) were used to investigate the potential correlation between chemical components and endothelial cell protection. The results indicated that BBTD could reduce ROS and hs-CRP levels in HUVEC cells, and the pharmacological activities in 18 batches of BBTD samples were significantly different. The results of BCA indicated that Gastrodin, Liquiritin, Hesperidin, Isoliquiritin, Hesperetin, and Isoliquiritigenin might be the active constituents to activate ROS and suppress hs-CRP as determined by spectrum-effect relationships. The antioxidant and anti-inflammatory activities of the 6 components at different concentration were verified, and the results showed that all of them had good antioxidant and anti-inflammatory activities in a concentration-dependent manner. This study showed that activity determination and spectral correlation can be used to search for active substances in Chinese medicine formula and provide data support for quality control of Traditional Chinese medicine (TCM).

## 1 Introduction

Banxia Baizhu Tianma decoction (BBTD), as a classical representative prescription, has the effects of expectorating phlegm, dispelling wind, invigorating the spleen and dispersing overflow. Based on TCM theory and clinical practice for many years, BBTD is especially suitable for the lack of exercise, abdominal obesity, and hypertension (Jiang et al., 2021). Recent studies have shown that BBTD has the potential to lower blood pressure *in vivo* and *in vitro* (Jiang J. et al., 2019). As reactive derivatives of oxygen metabolism, the activity of Reactive Oxygen Species (ROS) changes due to various stimuli, including G-protein coupled receptor agonists, growth factors, perfusion pressure, flow, and oxygen in vascular smooth muscle and endothelial cells. ROS are involved in smooth muscle contraction, endothelium-dependent relaxation, smooth muscle growth, proliferation, and migration, thereby contributing to fine-tuning of blood flow, arterial wall thickness, and vascular resistance (Knock, 2019). Oxidative stress will occur when the equilibrium state of ROS in the body is broken, which plays a key role in the development of vascular dysfunction under special conditions such as hypertension. It is characterized by NO loss bioavailability, vascular inflammation and endothelial dysfunction. Given that oxidative stress is a key regulating factor of vascular dysfunction, the application of classical antioxidant oxidation treatment seems to be a promising treatment for vascular disease, such as angiotensin converting enzyme (ACE) inhibitors or angiotensin AT1 receptor antagonist. It is confirmed that angiotensin II plays a key role in the reaction (Do Vale and Tirapelli, 2020). High sensitivity C-reactive protein (Hs-CRP) is a common biomarker of cardiovascular disease. Studies have shown that CRP directly involved in endothelial dysfunction in patients with high blood pressure, the development of vascular sclerosis and blood pressure, and CRP indicators can serve as a biomarker associated with atherosclerosis, vascular stiffness, end-organ injury, and the development of cardiovascular events (Hage, 2014; Zhang J. et al., 2019). CRP is an acute inflammatory protein that increases up to 1,000-fold at sites of infection or inflammation. There is now growing evidence that CRP plays an important role in inflammatory processes, and CRP is used as a classic marker of inflammation and cardiovascular events. Therefore, endothelial proliferation rate, ROS and Hs-CRP can be used as potential targets of antihypertensive effect of BBTD.

UPLC fingerprint is an effective tool to evaluate the quality of TCM, and identify the authenticity of traditional Chinese medicine as well as its components (Sun et al., 2019). It is a scientific method to clarify the substance basis of TCM pharmacological action and establish the quality control method of TCM to determine the correlation between fingerprint and biological activity by using spectrum-effect relationship (Farrag et al., 2019; Zhang J. et al., 2019; Liao et al., 2021). In this study, 18 BBTD samples were fingerprinted for component analysis, and the antioxidant and anti-inflammatory activities of endothelial cells were determined. Statistical methods were used to determine the relationship between spectrum and activity, identify possible active components, and further verify the activity.

## 2 Materials and Methods

### 2.1 Chemicals, Reagents and Materials

18 batches of BBTD comprising of Pinelliae Rhizoma (PR), Gastrodiae Rhizoma (GR), Rhizoma Atractylodis Macrocephalae (RAM), Citri Exocarpium Rubrum (CER), Poria (PO) and *Glycyrrhiza uralensis* Fisch (GUF) were included in this study. PR is the dry rhizome of Pinellia ternata (Thunb.) Makino [Araceae]. GR is the dry rhizome of Gastrodia elata Blume [Orchidaceae]. RAM is the dry rhizome of Atractylodes macrocephala Koidz [Compositae]. CER is the dry ripe peel of Citrus reticulata Blanco [Rutaceae]. PO is the dry sclerotia of Poria cocos (Schw.)Wolf [Polyporaceae]. GUF is the dried rhizome of *Glycyrrhiza* uralensis Fisch [Leguminosae]. The 18 batches of herbs came from Chinese herbal medicine markets in Anhui, Hebei, Shandong, Jilin, Guangxi, Beijing, Hunan, Sichuan and other places. All herbs were identified by Professor Jin Guangqian, TCM identification expert of Shandong Academy of Traditional Chinese Medicine. Each herb came from 3 different producing areas. L_18_ (3^7^) random number table method was used to randomly combine and sequence these 6 herbs, and 18 batches of BBTD samples (S1-S18) were obtained.

Acetonitrile (HPLC grade) and Methanol (HPLC grade) were purchased from Fisher Scientific Co. (FairLawn, NJ, United States). Ultrapure water was acquired from a Millipore Milli-Q-Plus system (Millipore, Bedford, MA, United States). The Gastrodin (a), Liquiritin (b), Narirutin (c), Naringin (d), Hesperidin (e), Neohesperidin (f), Isoliquiritin (g), Liquiritigenin (h), Hesperetin (i), Isoliquiritigenin (j), Nobiletin (k), Protopine (l), Atractylenolide III (m), Poricoic acid A (n) and Glycyrrhetinic acid (o) were purchased from Chengdu Skoqi-Biotechnology Co. Ltd. (Chengdu, China).

ROS kit was acquired from Solarbio Technology Co., Ltd. (Beijing, China). Hs-CRP enzyme-linked immunosorbent assay kit was purchased from Yubo Biotechnology Co., Ltd. (Shanghai, China). DMEM medium RPMI - 1640, fetal bovine serum, penicillin and streptomycin were purchased from biological sharp, from Shanghai, China. HUVEC cell line was purchased from Wuxi Bohe Biomedical Technology Co., LTD. (Jiangsu, China).

### 2.2 Instruments

Thermo Acquity UPLC system (Thermo, Waltham, MA, United States) consisted of a photodiode array (PDA)detector, autosampler manager, column compartment, and a binary solvent delivery pump and was connected to Thermo chromeleon software. Inverted photographic microscope was purchased from Tokyo, Japan. Enzyme label detector was purchased from Thermo (United States). palmitic acid was from MedChemExpress (United States). ELISA kits were purchased from BosterBio (United States). Dimethyl sulfoxide (DMSO) was acquired from Invitrogen (United States).

### 2.3 Ultra high Performance Liquid Chromatography Fingerprints

#### 2.3.1 Plant Sample Preparation

PR, GR, RAM, CER, PO and GUF was ground into powder using a mill. 4.6 g BBTD powder consisted of PR, GR, RAM, CER, PO and GUF powder (ratio 9:6:6:6:15:4) were dissolved in 50 ml water. The proportion of medicinal materials is mainly determined according to the ancient Chinese medicine book “Yi Xue Xin Wu”. The mixture was heated to boil and simmered for 1.0 h. After the sample was cooled, it was centrifuged at 3,000 rpm for 10min and then concentrated with a rotary evaporator.

#### 2.3.2 Preparation of Standard Compounds

All standard compounds were weighed accurately and dissolved in methanol to form reserve solution, which was stored at 4°C. All reserve fluids are diluted to the desired concentration and then mixed immediately prior to analysis. The final concentrations were 56.8 μg/ml(a), 38.9 μg/ml(b), 59.4 μg/ml(c), 59.9 μg/ml(d), 56.8 μg/ml(e), 40.5 μg/ml(f), 38.9 μg/ml(g), 15.7 μg/ml(h), 53.9 μg/ml(i), 31.8 μg/ml(j), 17.8 μg/ml (k), 18.6 μg/ml(l), 19.3 μg/ml(m), 71.8 μg/ml (n) and 59.2 μg/ml (o), respectively.

#### 2.3.3 Chromatographic Conditions

The solution was filtered by 0.22 μm microporous membrane and injected into UPLC for analysis. An Acclaim TM RSLC Lot Validation-120 C18 column (2.1 mm × 100 mm i. d., 2.2 μm particle size) (Thermo, Sunnyvale, CA, United States) was used for chromatographic separation. The temperature of the column temperature oven was set as 30°C. The mobile phase was a mixture of acetonitrile (solvent A) and water (solvent B) with a flow rate at 0.30 mlmin^−1^. Gradient elution condition: 0–5 min, 5–12% A; 5–20 min, 12%-26%A; 20–30 min, 26–80% A; 30–35 min, 80%–100% A. The absorption wavelength was set at 235 nm and the injection volume was 5.0 μL.

#### 2.3.4 Methodology Validation

The standard curve and linear range were established in the method validation part, and the precision, stability and repeatability were investigated. Precision was calculated by 6 consecutive injections of the same sample solution, and repeatability was evaluated by six samples from the same source. A sample solution was stored in a volumetric flask at room temperature and repeated injection analysis was performed within 1 day (0, 4, 8, 12, 16 and 24 h) to investigate the stability of the sample at room temperature.

#### 2.3.5 Peak Identification

The standard solution was injected into the UPLC system for qualitative analysis, and the retention time of the standard was recorded. The retention time of a-o was determined by comparing the retention time of reference substance.

#### 2.3.6 Fingerprint Established and Evaluated

For 18 batches of samples, each sample solution was injected three times according to the determined UPLC conditions, and fingerprint analysis was carried out. UPLC fingerprint data was saved in CDF format. Traditional Chinese medicine chromatographic fingerprint system (Version 2012A) was used to automatically match the chromatographic peak fingerprints, and then the chromatogram of different extracts was generally compared by using the median method to form reference chromatogram. The similarity between chromatogram and reference chromatogram of different extracts in 18 samples was calculated by using this software.

### 2.4 Cell Experiments

#### 2.4.1 Cell Viability Assay

HUVEC cells were cultured in the RPMI-1640 DMEM supplemented with 10% fetal bovine serum, penicillin (100 U/mL), and streptomycin (100 μg/ml) at 37°C under a humidified atmosphere containing 5% CO2. The mother liquor of 200 mM was obtained by dissolving 0.1024 g Palmitic acid in 20 ml DMSO, diluted to 200 umol/L with RPMI-1640 complete medium.All Eighteen water extracts of BBTD (1 mg/ml) were dissolved in the RPMI-1640. After incubation at 37°C with 5% CO2 for 24 h, HUVEC cells were inoculated on a 96-well plate for 24 h.

#### 2.4.2 Antioxidant Activity Test

Briefly, 200 μL of each extract solution under “2.4.1” was mixed with DCFH-DA (10 μmol L^−1^), then diluted to 1 ml. After stayed and reacted in dark for 20 min at 37°C, Using fluorescence inversion microscope to observe the fluorescence intensity of cells and take pictures. and converted to radical scavenging activity by the following equation:

Scavenging activity(%)=(Amodel-Asample)/(Amodel-Ablank)×100%

Serum-free medium (supplement to the total volume of 6 ml) plus sample solution (200 μL)/DCFH-DA solution (4 ml) was used as a blank/control. Each sample was run in triplicate and averaged.

#### 2.4.3 High Sensitivity C-Reactive Protein Inhibition Rate Test

Hs-CRP value was assessed by absorbance determination using an enzyme-linked immunosorbent assay (ELISA) kit. The hs-CRP concentration in Cell supernatant was determined by ELISA kit, then the absorbance was measured at 450 nm using a microplate reader. The inhibition of hs-CRP release was calculated according to the following:

hs-CRP inhibition rate(%)=(Cmodel-Csample)/(Cmodel-Cblank) ×100%

The experiments were performed in triplicates, the data were presented as the mean ± SD.

### 2.5 Hierarchical Cluster Analysis

As a multivariate analysis method, Hierarchical Cluster Analysis (HCA) sorted specimens into clusters. This analysis method could maximize each cluster in homogeneity, maximized the heterogeneity between cluster at the same time, the objects were divided into specific cluster (Song et al., 2019; Jia et al., 2021). This study applied HCA to assess the correlation of 18 samples of BBTD UPLC fingerprint, and used Heatmapper (http://www.heatmapper.ca/) for testing.

### 2.6 Bivariate Correlation Analysis

Pearson’s correlation coefficient was used to quantify the degree of co-location between paired data. In this study, the Bivariate correlation analysis function of SPSS statistical software (SPSS for Windows 20.0, SPSS Inc., United States) was used to analyze the correlation between the peak area value of UPLC fingerprint and the antioxidant/anti-inflammatory effect of HUVEC cells.

## 3 Results

### 3.1 Method Validation for Fingerprint

The relative standard deviations (RSD) of precision and reproducibility were both less than 2.60%, and samples for UPLC determination were stable at room temperature for 24 h.

### 3.2 Ultrahigh Performance Liquid Chromatography Fingerprints

**FIGURE 1:**
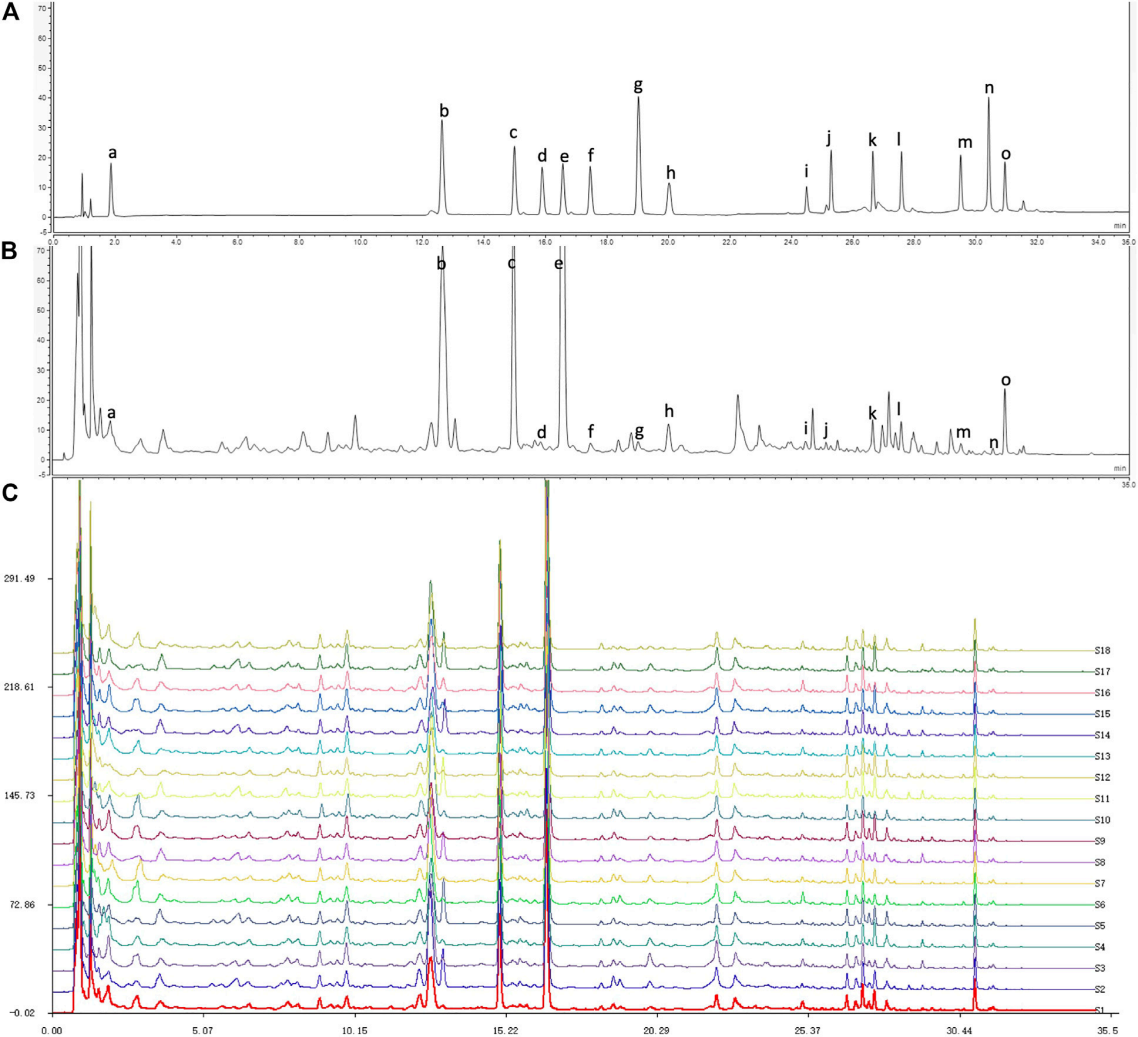
UPLC-PDA chromatogram of reference substances **(A)**, test sample **(B)** and 18 batches of BBTD **(C)**. 15 peaks were identified by comparison with standard substances: Gastrodin (a), Liquiritin (b), Narirutin (c), Naringin (d), Hesperidin (e), Neohesperidin (f), Isoliquiritin (g), Liquiritigenin (h), Hesperetin (i), Isoliquiritigenin (j), Nobiletin (k), Protopine (l), Atractylenolide III (m), Poricoic acid A (n) and Glycyrrhetinic acid (o).

18 bathes of BBTD samples were analyzed with UPLC. was shown in Figure 1. Mixed reference substances (Figure 1A), BBTD test sample (Figure 1B) and Chromatograms of all batches of BBTD (Figure 1C), were generated under the determined chromatographic condition. The similarity between reference fingerprints and chromatograms among 18 batches of BBTD samples was evaluated by calculating correlation coefficients. Similar chemical signatures were found across batches, and 15 common peaks were found over intervals of 1–30 min by comparing the UV spectra and UPLC retention times of 18 chromatograms. 15 common peaks were identified as Gastrodin (a), Liquiritin (b), Narirutin (c), Naringin (d), Hesperidin (e), Neohesperidin (f), Isoliquiritin (g), Liquiritigenin (h), Hesperetin (i), Isoliquiritigenin (j), Nobiletin (k), Protopine (l), Atractylenolide III (m), Poricoic acid A (n) and Glycyrrhetinic acid (o) by comparison. Peak a has the largest peak area in the fingerprint. As shown in Table 1, the peak areas of common peaks of different samples were different to some extent, and the RSD% of all common peaks were greater than 10%, indicating that the content of chemical components in different batches of BBTD samples from different producing areas were different.

**TABLE 1:** The relative peak area of 15 common peaks measured by UPLC-PDA.

Sample	Peak area of each compound														
	a	b	c	d	e	F	g	h	i	j	k	l	m	n	o
S1	2.9705	6.7800	6.9694	0.4316	19.4671	0.1729	0.2999	0.3552	0.1285	0.1413	0.7508	1.0291	0.1982	0.0848	1.4926
S2	3.4193	13.9931	7.9431	0.3181	23.5922	0.2120	0.7751	0.7416	0.1383	0.1564	0.7503	0.9441	0.2109	0.0978	1.0891
S3	3.4786	10.4605	9.5405	0.4705	25.6195	0.1619	0.5068	1.6416	0.1558	0.1739	0.7108	0.3911	0.1061	0.0793	1.4765
S4	1.2530	6.4111	9.2160	0.6422	14.9526	0.4228	0.5212	1.8039	0.1612	0.0809	0.7050	1.3536	0.1961	0.1335	1.4787
S5	2.9468	6.0807	9.3892	0.7715	16.6287	0.1772	0.3497	1.4001	0.1154	0.1202	0.7718	1.0118	0.2129	0.0962	1.5332
S6	4.0820	12.0234	8.1739	0.3407	18.3150	0.2274	0.6982	0.6319	0.1200	0.1141	0.6238	0.5591	0.1495	0.1675	1.4383
S7	3.6789	11.8086	7.9348	0.3843	17.7325	0.2395	0.5949	0.6166	0.1702	0.1542	0.6610	0.7323	0.1205	0.0843	1.3763
S8	2.0217	7.3211	8.1127	0.6066	18.4095	0.1055	0.3298	0.8529	0.0955	0.1492	0.6376	0.4799	0.1260	0.0771	1.3693
S9	1.8181	11.0485	9.5243	0.9790	25.2465	0.1254	0.3457	0.4269	0.1126	0.1531	0.9428	1.3085	0.2763	0.0939	1.0119
S10	3.0666	13.5824	9.7096	0.8454	24.4846	0.0748	0.5487	1.4372	0.1011	0.1663	0.6429	0.4886	0.1301	0.1070	1.3618
S11	3.5990	8.2719	7.7308	0.5255	16.9096	0.2513	0.3704	0.8104	0.1525	0.1340	0.8455	1.1785	0.2422	0.0961	1.3550
S12	1.1075	5.6802	9.5294	0.2282	19.5106	0.1747	0.3094	0.4383	0.0857	0.1281	1.0632	1.1950	0.1856	0.1488	1.3598
S13	3.9153	6.9918	8.4033	0.5787	22.2395	0.1761	0.3595	0.7494	0.1256	0.2233	0.5665	0.7138	0.1363	0.0913	1.3832
S14	1.4633	5.1123	7.8654	0.2456	19.2884	0.2443	0.2840	1.3744	0.2460	0.1398	0.7797	0.4300	0.1348	0.1302	1.4210
S15	3.8311	10.4729	10.0549	0.5285	21.2086	0.3013	0.5395	1.0647	0.1683	0.1490	0.7798	1.3745	0.2739	0.0901	1.4228
S16	3.5314	6.4247	9.5296	0.3500	19.2174	0.1608	0.3582	0.4160	0.1672	0.1042	0.8654	0.5053	0.1157	0.1203	1.0202
S17	2.4918	11.8817	9.4389	0.4328	25.6747	0.1411	0.5792	0.7563	0.2447	0.1280	0.7337	1.4214	0.2626	0.0977	1.4113
S18	1.9341	5.1496	8.0547	0.5038	19.5670	0.1564	0.2901	0.4407	0.1086	0.1158	0.6136	0.7767	0.1513	0.1297	1.4159
RSD%	34.85	34.03	10.32	39.86	16.80	40.63	33.59	51.65	31.31	22.03	16.68	41.07	31.90	23.91	11.39

### 3.3 Fingerprint Similarity

In order to assess the similarity between these samples, fingerprints of 18 BBTD samples were compared and analyzed with reference fingerprints. The similarity between the fingerprint of each sample of different samples and the reference fingerprint ranges from 0.897 to 0.992, as shown in Table 2.

**TABLE 2:** Similarities of different batches BBTD samples from various regions.

Sample	Similarities	Sample	Similarities	Sample	Similarities
S1	0.989	S7	0.964	S13	0.992
S2	0.897	S8	0.992	S14	0.967
S3	0.988	S9	0.991	S15	0.986
S4	0.982	S10	0.974	S16	0.989
S5	0.987	S11	0.984	S17	0.983
S6	0.920	S12	0.990	S18	0.984

### 3.4 Antioxidant Activity Test

**FIGURE 2:**
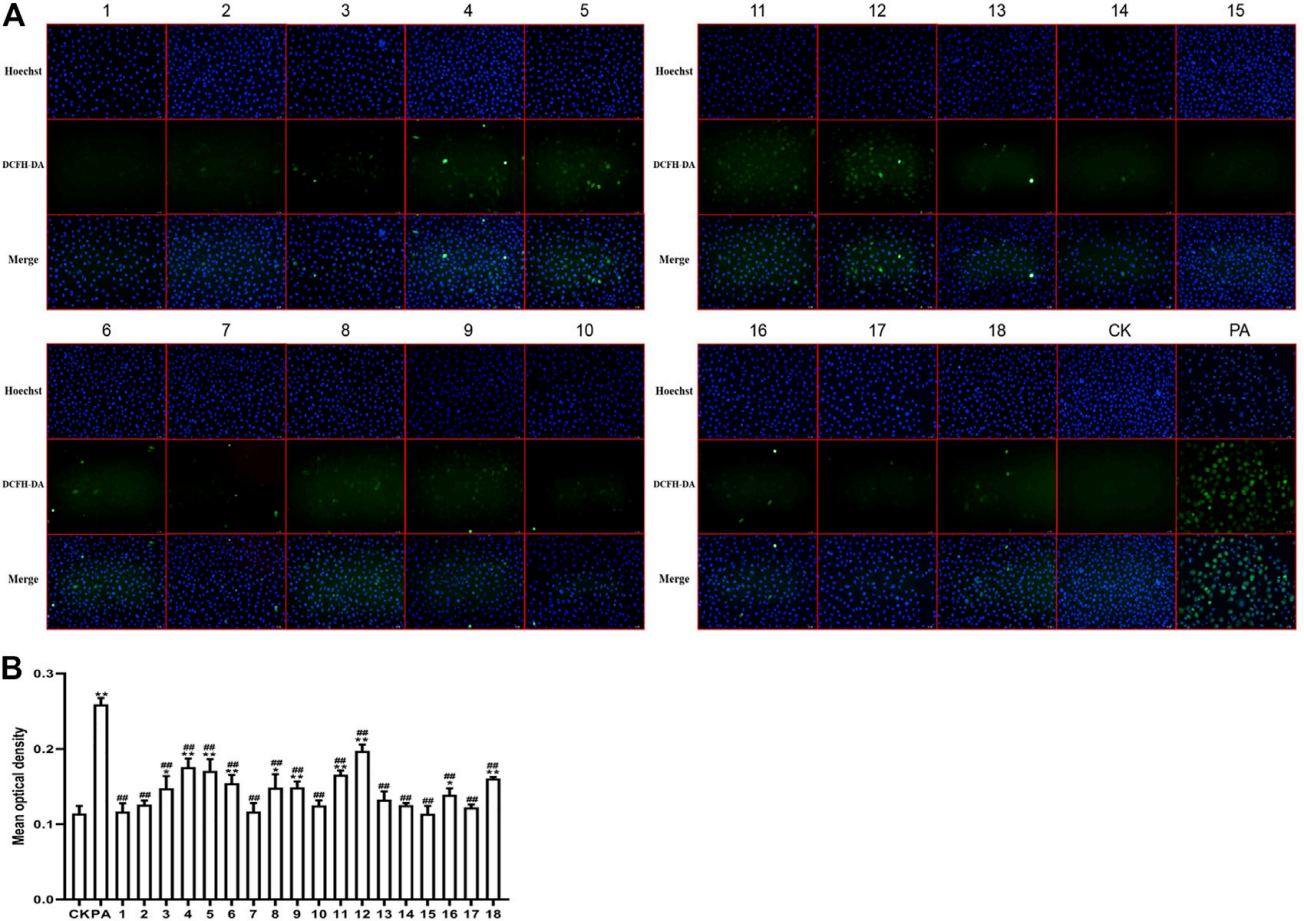
ROS fluorescence intensity **(A)** and scavenging activity **(B)** of Eighteen batches BBTD samples.

The ROS inhibition rates of 18 BBTD samples was analyzed by ANOVA and further multiple comparisons were performed. All the model results showed *p* = 0.000 < 0.01, indicating that there were significant differences in ROS scavenging ability of different extracts. ROS fluorescence intensity and inhibition rates of 18 batches of BBTD extracts were shown in Figure 2 and Table 3.

**TABLE 3:** ROS inhibition rates of 18 batches of BBTD extracts (mean ± SD, *n* = 3).

Sample	Mean optical density	Scavenging rate %	Sample	Mean optical density	Scavenging rate %
CK	0.1141 ± 0.0102	—	S9	0.1489 ± 0.0080	196.77
PA	0.2539 ± 0.0084	—	S10	0.1250 ± 0.0065	217.68
S1	0.1166 ± 0.0110	225.03	S11	0.1656 ± 0.0057	182.11
S2	0.1258 ± 0.0056	217.03	S12	0.1975 ± 0.0083	154.17
S3	0.1476 ± 0.0163	197.87	S13	0.1323 ± 0.0110	211.29
S4	0.1758 ± 0.0115	173.17	S14	0.1253 ± 0.0027	217.41
S5	0.1711 ± 0.0153	177.33	S15	0.1136 ± 0.0106	227.68
S6	0.1543 ± 0.0112	192.00	S16	0.1392 ± 0.0085	205.29
S7	0.1168 ± 0.0111	224.90	S17	0.1222 ± 0.0041	220.12
S8	0.1485 ± 0.0179	197.14	S18	0.1609 ± 0.0018	186.21

### 3.5 High Sensitivity C-Reactive Protein Inhibition Rate Test

**FIGURE 3:**
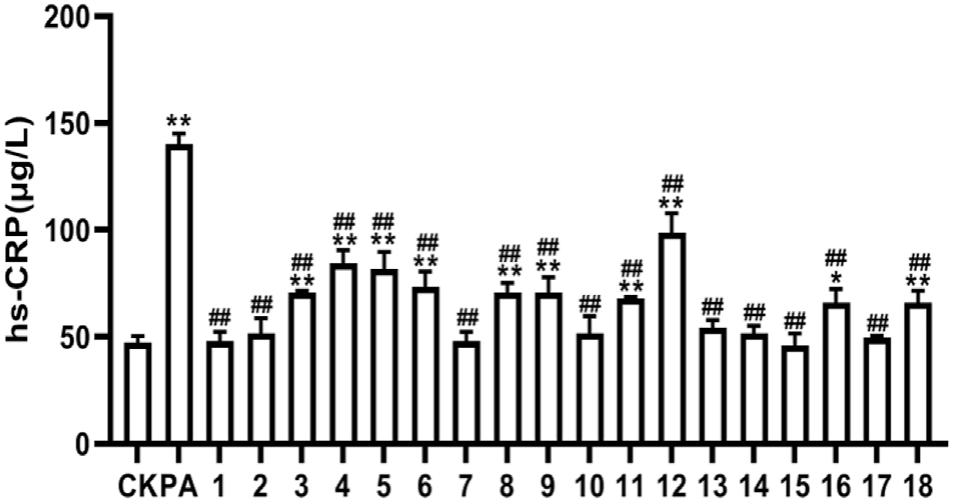
Hs-CRP concentration of 18 batches among BBTD samples.

Then the hs-CRP inhibition rate in 18 BBTD samples was analyzed by ANOVA model and multiple-comparison model, and the resulted demonstrated that all has significant difference (*p* = 0.001 < 0.05). The hs-CRP concentration and inhibition rate of BBTD extracted from 18 batches were shown in Figure 3 and Table 4.

**TABLE 4:** The hs-CRP inhibition rate of BBTD extracted from 18 batches (mean ± SD, *n* = 3).

Sample	Mean optical density	Scavenging rate %	Sample	Mean optical density	Scavenging rate %
CK	47.3030 ± 3.1926	—	S9	70.6364 ± 7.2727	31.83
PA	140.0303 ± 5.0069	—	S10	51.5455 ± 8.1818	46.54
S1	47.9091 ± 4.5455	48.65	S11	67.9091 ± 0.9091	34.41
S2	51.5455 ± 7.2727	46.54	S12	98.8182 ± 9.0909	22.72
S3	70.6364 ± 0.9091	31.83	S13	54.2727 ± 3.6364	44.84
S4	84.2727 ± 6.3636	15.16	S14	51.5455 ± 3.6364	46.54
S5	81.5455 ± 8.1818	19.12	S15	46.0909 ± 5.4545	49.65
S6	73.3636 ± 7.2727	29.05	S16	66.0909 ± 6.3636	36.02
S7	47.9091 ± 4.5455	48.65	S17	49.7273 ± 0.9091	47.62
S8	70.6364 ± 4.5455	31.83	S18	66.0909 ± 5.4545	36.02

### 3.6 Results of Hierarchical Cluster Analysis

**FIGURE 4:**
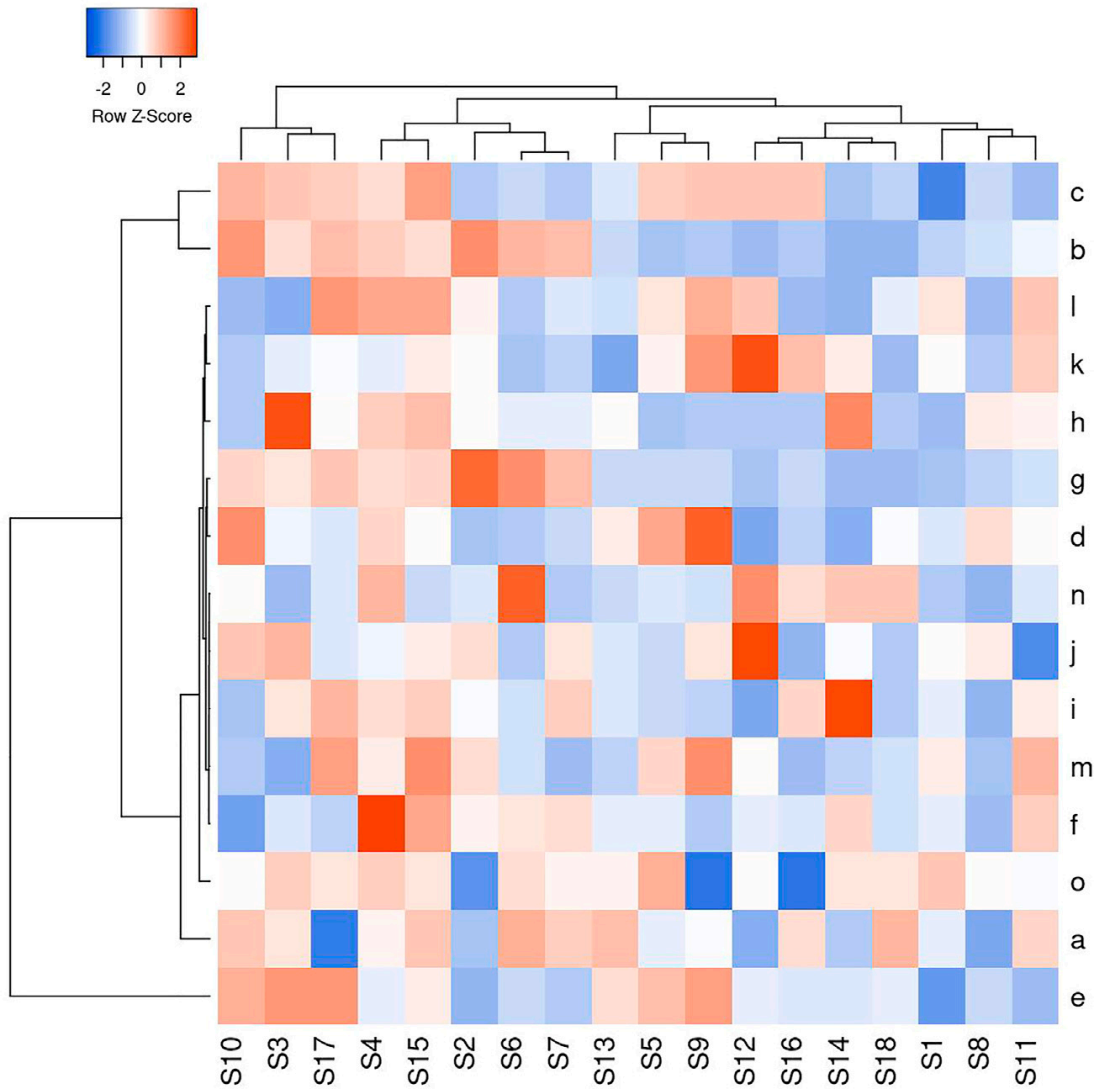
HCA heat map for 18 BBTD samples and 15 chemical compounds.

Heat map is a graphical representation of data, each color unit corresponding to the matrix contains a single value. In the vicinity of HCA, 15 common peaks of 18 BBTD samples are shown by heat maps, and three clusters can be detected from Figure 4. Cluster 1 was composed of S3, S10 and S17, cluster 2 consisted of S2, S4, S6, S7, and S15, and cluster 3 consisted of S1, S5, S8, S9, S11, S12, S13, S14, S16, and S18. The results showed that the different sources of BBTD samples with similar chemical fingerprint, but the peak area of the ingredients are different. HCA can achieve preliminary separation of the samples at the chemical level. The different contents of chemical components in the cluster samples indicate that the chemical components may be different according to different places of origin.

### 3.7 Spectrum-Effect Relationship Analysis

**FIGURE 5:**
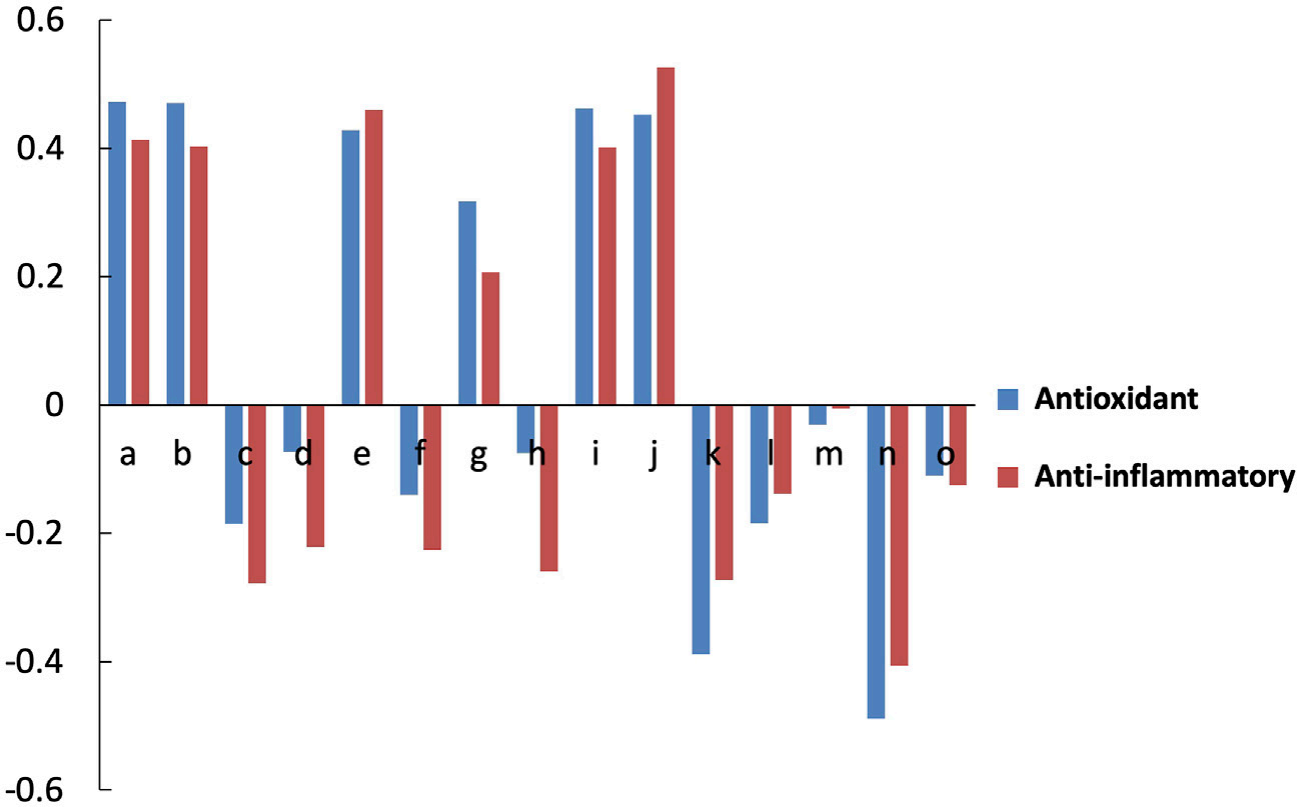
Analysis of the spectrum-effect relationship: the correlation coefficient between the content of chemical compounds and bioactivity.

As shown in Figure 5 and Table 5, the correlation coefficient showed that 6 peaks a, b, e, g, i, and j were positively correlated with activity. a, b, e, g, i and j were identified as Gastrodin, Liquiritin, Hesperidin, Isoliquiritin, Hesperetin and Isoliquiritigenin, respectively.

**TABLE 5:** The correlations coefficient of Multivariate Statistical Analysis.

	Correlations and grade between peak and antioxidant	Correlations and grade between peak and anti-inflammatory
A	0.472^*^	0.413
B	0.471^*^	0.403
C	−0.185	−0.278
D	−0.073	−0.221
E	0.428	0.460
F	−0.140	−0.226
G	0.317	0.207
H	−0.075	−0.259
I	0.462	0.401
J	0.452	0.526^*^
K	−0.388	−0.273
L	−0.184	−0.138
M	−0.031	−0.005
N	−0.489^*^	−0.406
O	−0.110	−0.125

### 3.8 Experimental Validation

**FIGURE 6:**
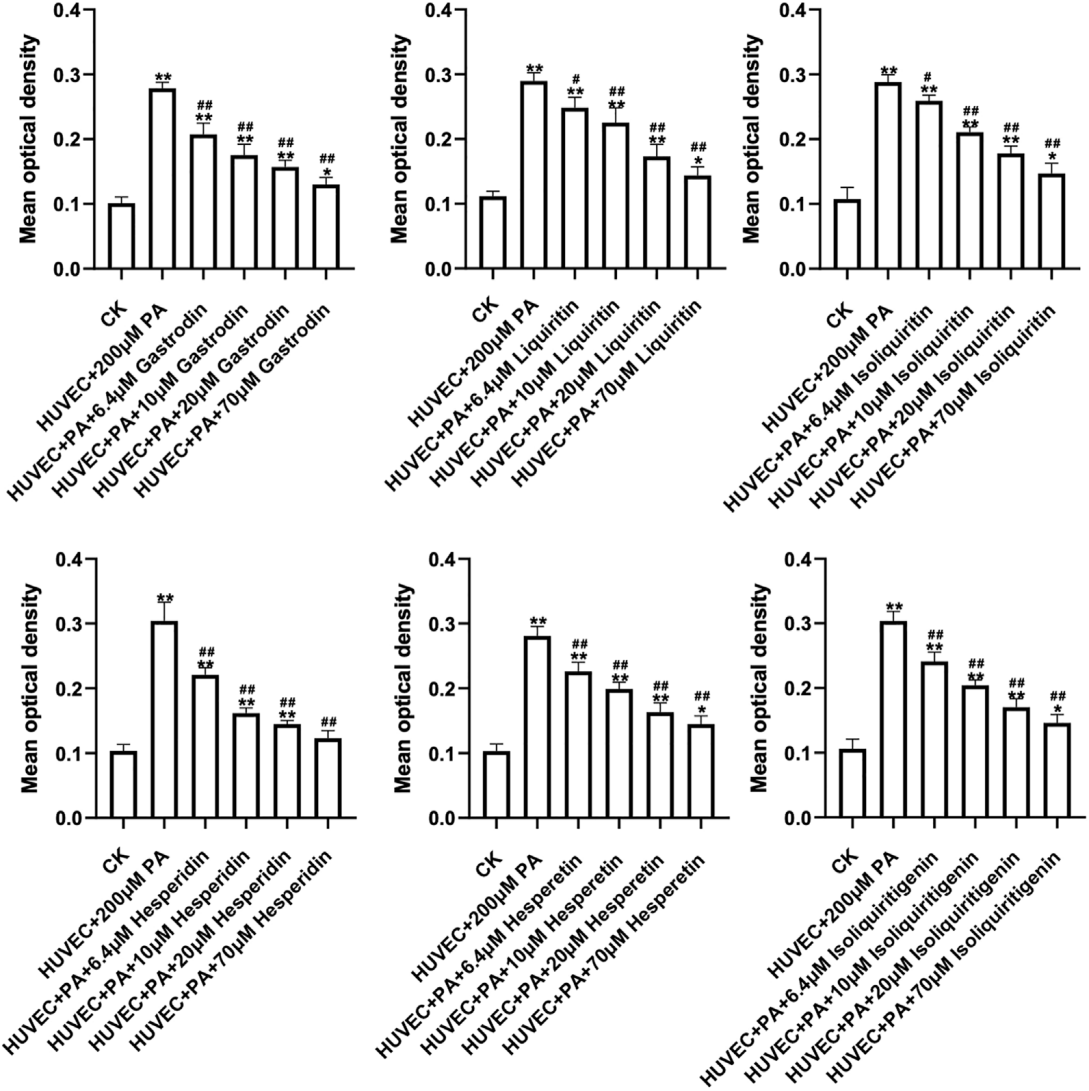
Concentration-response curves of 6 constituents Antioxidant effects.

**FIGURE 7:**
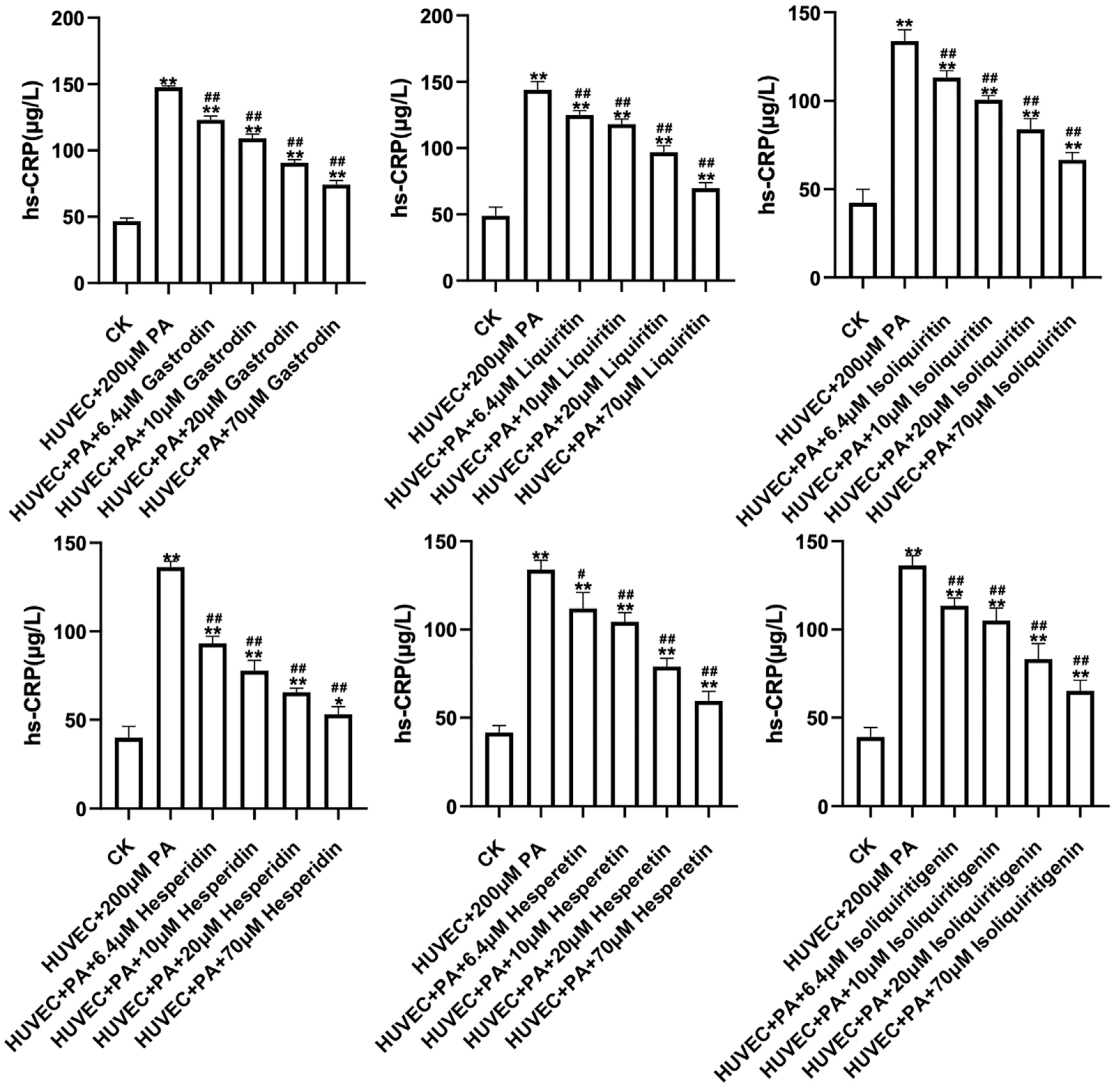
Concentration-response curves of 6 constituents Anti-Inflammatory effects.

6 correlated peaks, Gastrodin (a), Liquiritin (b), Hesperidin (e), Isoliquiritin (g), Hesperetin (i) and Isoliquiritigenin (j) were positively correlated with antioxidant and anti-Inflammatory activity of HUVEC cells. In order to verify the protective effect of the 6 components on HUVEC cells, the antioxidant and anti-inflammatory effects of the 6 components on HUVEC cells were measured at different concentrations. The results showed that all samples could promote the proliferation of HUVEC cells in a concentration-dependent manner (Figures 6, 7).

## 4 Discussion

Traditional Chinese medicine decoction is a kind of oral liquid preparation made by decocting several Chinese herbs in proper dosage proportion according to the theory of “Jun-Chen-Zuo-Shi” (Li et al., 2021a). Based on accurate diagnosis of “looking, listening, asking and feeling the pulse” of patients, TCM often selects prescriptions according to the physical signs of patient and changes in the course of disease. Combined with the “taste and meridian return” of drugs, constantly adjusts them in the process of practice to accumulate experience. Therefore, the prescription varies with the person, the disease and the progress of disease, and the prescription is adjusted and modified at any time, which fully embodies the characteristics of personalized treatment (Jiang L. et al., 2019). TCM and western medicine come from different cultural backgrounds, theoretical systems and medical models are greatly different. Under the guidance of modern medical theory, Western medicine is researched and developed for some pathological process of diseases or symptoms. Most of them are single chemical components, with clear targets and mechanisms of action and clear adverse reactions. Traditional Chinese medicine has become a hot spot in the research field of traditional Chinese medicine decoction because of its many producing areas and difficult quality control (Kang et al., 2019).

Many studies have shown that the study of spectrum-effect relationshipship is an effective method to control the quality of traditional Chinese medicine, because it can be used as a chromatographic fingerprint with biological activity and explore the quality of markers related to clinical efficacy (Fan et al., 2020). Traditional Chinese medicine is the product of multi-component and multi-target synergy. It is impossible to control the quality of traditional Chinese medicine only by the content of one characteristic ingredient. Fingerprint has integrity and fuzziness, which is well adapted to the complexity and diversity of TCM components and can evaluate and control the quality of TCM and its compounds (Zhang H. et al., 2019; Liu et al., 2020). Therefore, in this study, we constructed fingerprints of 18 batches of medicinal materials, and searched for common peaks as potential active ingredients.

This study found that BBTD can protect vascular endothelial cells, promote endothelial cell proliferation, reduce ROS and Hs-CRP levels, and indicated that Gastrodin (a), Liquiritin (b), Hesperidin (e), Isoliquiritin (g), Hesperetin 1) and Isoliquiritigenin (j) may be the main active components for promoting the proliferation of HUVEC cells. These results suggest that BBTD may reduce oxidative stress and inflammatory expression of endothelial cells through these 6 components, and play a protective role in endothelial cells, thus achieving the effect of reducing hypertension.

6 correlated peaks, Gastrodin (a), Liquiritin (b), Hesperidin (e), Isoliquiritin (g), Hesperetin (i) and Isoliquiritigenin (j) were positively correlated with antioxidant and anti-Inflammatory activity of HUVEC cells. In order to verify the protective effect of the 6 components on HUVEC cells, the antioxidant and anti-inflammatory effects of the 6 components on HUVEC cells were measured at different concentrations.

It can be seen from the results that all the six potential active ingredients (Gastrodin, Liquiritin, Hesperidin, Isoliquiritin, Hesperetin and Isoliquiritigenin) have antioxidant and anti-inflammatory activities to varying degrees, which is consistent with the results of literature studies. Zhang H et al. found that Gastrodin induced HO-1 and Nrf2 up-regulation to alleviate H_2_O_2_-induced oxidative stress in mouse liver sinusoidal endothelial cells through p38 MAPK phosphorylation (Zhang et al., 2018). Ye T et al. discovered that Gastrodin attenuates the diabetic encephalopathy by inhibiting ER stress and NLRP3 inflammasome activation (Ye et al., 2018). Application of Liquiritin to exposed skin of rats can reduce the increase in ROS, pro-inflammatory factors, and MMPs caused by UVB irradiation and increased the levels of Sirtuin3 (SIRT3) and Collagen *α*1 (Li et al., 2021b). Hesperidin and Hesperetin can decreased inflammatory mediators and exerted significant antioxidant effects (Tejada et al., 2018; Muhammad et al., 2019). Isoliquiritin can protect the kidney of membranous glomerulonephritis model rats through antioxidant and anti-inflammatory effects, and these effects were found to be related to the activation of Nrf2 and the down-regulation of NF-κB pathway (Liu et al., 2019). Isoliquiritigenin is also used in the prevention and treatment of a variety of diseases because of its anti-inflammatory and antioxidant properties (Peng et al., 2015). Therefore, we hypothesized that these six chemical components could serve as potential quality markers for BBTD protection of endothelial cells.

The results of this study suggest that the spectrum-effect relationship can provide an effective tool for the correlation between the quality indexes of TCM decoction and clinical efficacy, and also provide an example for the quality control of other TCM decoction. However, these 6 compounds cannot be used as an indicator of overall quality control of BBTD because antioxidant and anti-inflammatory effects are only two of its many clinical effects. In addition, due to instrument limitations, we mainly focused on compounds with strong UV signals (PDA detector). Some of the components may play an important role in antioxidant and anti-inflammatory effects. However, they were not detected in this study due to their low levels. Therefore, further studies are needed to determine whether Gastrodin, Liquiritin, Hesperidin, Isoliquiritin, Hesperetin and Isoliquiritigenin can be used as a quality control indicator for the treatment of other diseases, as well as a quality control indicator for whether there are other potential active ingredients in BBTD. Next, this study will consider mass spectrometry analysis, so as to explore more active ingredients or metabolites.

## 5 Conclusion

UPLC fingerprints of 18 different BBTD extracts are established by UPLC-PDA technique. The results show that the extracts of BBTD has antioxidant and anti-inflammatory activities, significantly increases the proliferation of endothelial cells and protected endothelial cells. The spectrum-effect relationship between fingerprint and activity value (ROS inhibition rate and Hs-CRP inhibition rate) is established by multivariate statistics. The results shows that Gastrodin, Liquiritin, Hesperidin, Isoliquiritin, Hesperetin, and Isoliquiritigenin have antioxidant and anti-inflammatory activities to different degrees. These six ingredients can be used as potential pharmacodynamic substances of BBTD to protect endothelial cells.

## Data Availability

The original contributions presented in the study are included in the article/Supplementary Material, further inquiries can be directed to the corresponding author.
